# Dinucleotide Weight Matrices for Predicting Transcription Factor Binding Sites: Generalizing the Position Weight Matrix

**DOI:** 10.1371/journal.pone.0009722

**Published:** 2010-03-22

**Authors:** Rahul Siddharthan

**Affiliations:** The Institute of Mathematical Sciences, Chennai, Tamil Nadu, India; Memorial Sloan Kettering Cancer Center, United States of America

## Abstract

**Background:**

Identifying transcription factor binding sites (TFBS) *in silico* is key in understanding gene regulation. TFBS are string patterns that exhibit some variability, commonly modelled as “position weight matrices” (PWMs). Though convenient, the PWM has significant limitations, in particular the assumed independence of positions within the binding motif; and predictions based on PWMs are usually not very specific to known functional sites. Analysis here on binding sites in yeast suggests that correlation of dinucleotides is not limited to near-neighbours, but can extend over considerable gaps.

**Methodology/Principal Findings:**

I describe a straightforward generalization of the PWM model, that considers frequencies of dinucleotides instead of individual nucleotides. Unlike previous efforts, this method considers *all* dinucleotides within an extended binding region, and does not make an attempt to determine *a priori* the significance of particular dinucleotide correlations. I describe how to use a “dinucleotide weight matrix” (DWM) to predict binding sites, dealing in particular with the complication that its entries are not independent probabilities. Benchmarks show, for many factors, a dramatic improvement over PWMs in precision of predicting known targets. In most cases, significant further improvement arises by extending the commonly defined “core motifs” by about 10bp on either side. Though this flanking sequence shows no strong motif at the nucleotide level, the predictive power of the dinucleotide model suggests that the “signature” in DNA sequence of protein-binding affinity extends beyond the core protein-DNA contact region.

**Conclusion/Significance:**

While computationally more demanding and slower than PWM-based approaches, this dinucleotide method is straightforward, both conceptually and in implementation, and can serve as a basis for future improvements.

## Introduction

Transcription factors (TFs) are proteins that regulate transcription, the process by which messenger RNA is synthesised from a DNA template. TFs facilitate or inhibit recruitment of the RNA polymerase by binding to DNA, usually near the gene that they regulate. Their binding sites are short nucleotide patterns or “motifs”. Detection of such motifs in DNA sequence is therefore of great practical importance in the study of gene regulation. These motifs are not exact strings: while most binding sites for a given factor resemble a “consensus string” (for example, ACGCGT, the most common binding sequence for the MBP1 protein in budding yeast), mismatches and variations often occur.

An early study of the variability and statistical properties of binding sites was by Berg and von Hippel [1]. The most popular representation of binding sites is the position weight matrix (PWM) [2], [3], which has a convenient visual depiction, the sequence logo [4]. For a motif of length 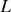, a PWM is a 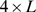 matrix, 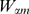, where 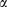 is A, C, G or T, and 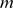 is an integer ranging over the length 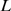 of the binding sequence. 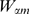 is the probability of seeing nucleotide 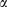 at position 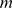; the sum over 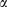, for each 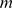, is unity. Typically, a PWM is estimated by aligning a large number of known binding sites, and calculating the relative frequencies of each nucleotide at each position. A “pseudocount” is generally added to the raw nucleotide counts, to allow for the limited size of the data. Thus, given 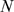 aligned sequences, where the number of nucleotides of type 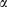 at column 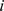 is 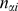 (with 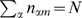 for all 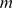), the weight matrix is given by
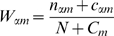
(1)where 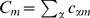. We choose 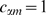, which corresponds to a “uniform prior” or complete lack of prior bias (formally, a pseudocount is equivalent to assuming a Dirichlet prior: see Materials and Methods for further discussion). A sequence logo [4] is a visual representation where the four possible nucleotides are stacked at each position 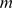, one atop the other, with their relative heights proportional to their weights in the 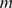
′th PWM column, and the total height proportional to the “information content” of the PWM column, defined as 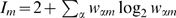.

A PWM assumes independence among different “columns” (values of 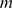). As an extreme example, it cannot describe a case where two successive positions contain the nucleotides AA or TT equally often but not AT or TA: a weight matrix will contain 0.5 for each of A and T at each position, and will imply that all four of AA, AT, TA and TT are equally probable. For the most part, such strong correlations are not observed among different nucleotides in binding sites, but it is known [5]–[7] that different sites are not completely independent. Nevertheless, Benos *et al.* [8], argued that the independent approximation is a good one in practice.

A related question is whether the binding *energy* can be written as a sum of single-nucleotide binding energies. Djordjevic *et al.* [9] argued that even with the additivity assumption for the binding energy (which they make), the binding probability should be modelled by a Fermi-Dirac function and not a Boltzmann function, while only the latter (which is the rare-binding limit of the former) can justify the PWM model. However, van Nimwegen *et al.* [10] (supporting text) use a simple maximum-entropy argument to show that the additivity assumption on energy does imply the PWM model for binding sites, if one also makes the reasonable assumption that binding sites have a significantly higher expected binding energy than random sites. Therefore, non-independence of nucleotide distributions in different positions probably implies non-additivity of the binding energy.

Several attempts have been made to go beyond PWMs. A biophysical model was presented by Djordjevic *et al.* [9], while several authors have considered purely statistical/bioinformatic approaches that take account of correlations (or other forms of binding-site heterogeneity not describable by PWMs) in various ways [11]–[14]. Recently, Sharon *et al.* [14] described a “feature-based” model that enhances the PWM picture with representations of other sequence features, including interdependencies in binding site positions). However, none of these approaches has achieved significant popularity, perhaps because they lack the conceptual simplicity of the PWM.

If the independence assumption is adequate, are nearest-neighbour dinucleotides sufficient? Theoretically, the question is made complicated by the effect of sequence on DNA conformation and bendability, which means that the DNA-protein contact interactions (which, one would expect, are reasonably local) are not the only factor at play. O'Flanagan *et al.* [15] observe contributions primarily from nearest-neighbour dinucleotides. However, Faiger *et al.* [16] report that some TATA boxes (binding sites for the TBP) have context-dependent conformations that require one to go beyond nearest-neighbour non-additivity. Sharon *et al.* [14] consider “features” that are much more complicated than nearest-neighbour dinucleotides. Below (see Results), we examine binding sites in yeast for several transcription factors, and conclude that dinucleotide correlations are significant in several cases, and occur with gaps of all lengths in a binding region, not just with nearest-neighbours.

In fact, it has been known for many years that DNA, particularly non-coding DNA, exhibits long-range power-law correlations [17], for reasons that remain unclear. Therefore, such correlations would not be surprising in binding sites.

A notable case where PWMs appear to be severely inadequate is the binding affinity of nucleosomes. Segal *et al.* [18] used dinucleotide matrices to model nucleosome-binding DNA sequences, but their approach differs significantly from what is described below: notably, they confine themselves to nearest-neighbour dinucleotides. I do not address nucleosomes here, but hope to do so at a future date.

Here I describe a straightforward extension of the PWM method, which reduces to the PWM representation for independent positions. Analogous to a position weight matrix 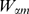, which gives the probability of observing each nucleotide 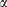 at each position 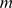, let us define 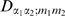, a *dinucleotide weight matrix* (DWM) that gives the probability of observing each pair of nucleotides 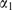 and 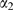 at each pair of positions 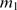 and 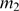 in a binding site. All pairs of positions are considered: recognising that correlations occur at all scales, we are not restricted to nearest-neighbours (as in [18]), and don't explicitly search for correlated pairs or features (as in [14]).

Defining such an object is easy: but the use of 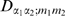 is not as straightforward as using 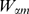 in predicting binding sites, because dinucleotide probabilities for different pairs of positions are not independent. With PWMs, one is interested in the likelihood 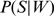 of observing the sequence 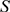 given a weight matrix model 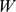; or the log-likelihood ratio 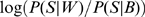 of observing the sequence given 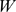, to observing it given a background model 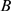. These numbers are readily calculated given the PWM and a simple background model: for example, if each nucleotide in the background model is represented by its actual genomic frequency (the model that is actually used throughout this work), 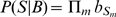 where 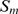 is the nucleotide at position 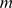 in the sequence, and 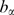 is the background probability of 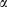. Meanwhile, 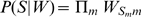, that is, the product of the weight matrix value for each nucleotide at each position in the sequence. Often, instead of a PWM, a log-odds matrix is used whose entries, when summed, directly yield the log-likelihood ratio (the matrices from yeast ChIP data [19], [20], that we use below, are in this format).

No such factorisation is possible for 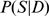, the probability of observing a sequence given a dinucleotide model. However, I introduce here a conceptually straightforward approximation. This is a Bayesian estimate of the posterior probability of each nucleotide at each position 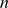, given the neighbouring sequence (ie, all nucleotides within the putative binding region at all positions 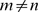. The product of these posterior probabilities, over all nucleotides, is treated as the likelihood of the sequence; and the log-odds is calculated as usual. The formula reduces, as it should, to the PWM value for any position 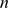 if nucleotides at other positions are independent of the nucleotide at 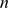. The formula is derived in Materials and Methods.

There are three complications with this approach, which may account for why such unrestricted DWMs have not been previously used: but the first two are answered here, and I argue that the third is an acceptable price to pay for the increased power.

First, there is the question of how to calculate with joint probabilities, or conditional probabilities, that are not independent. This is answered above; the method should in fact be more widely applicable, and this will be explored in the future.

Second, reliable estimation of 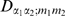 requires availability of many more sequences than estimation of 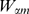, because there are only 4 nucleotides but 16 dinucleotides. But this is increasingly less of a problem, since dozens of known binding sites now exist for several factors across different species. In fact, based on the benchmark results below, I argue that this approach would be particularly useful in analysing binding data from high-throughput experiments (ChIP-chip or ChIP-seq): these yield thousands of putative binding sites, of which hundreds may be sufficiently high-confidence for this purpose. Details on how to estimate the DWM are in Materials and Methods.

Third, a DWM is a much larger object than a PWM: for a binding sequence of length 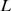, a PWM is 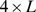-dimensional, while a DWM is 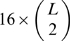 dimensional. The storage required is quadratic in 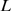. This is exacerbated by one of the key observations below: flanking sequence of several nucleotides improves predictions and appears to play a role in determining binding sites, even when only a “core motif” is prominent in a sequence logo. Therefore, though a PWM for eukaryotic factors is typically between 6 and 15 bp long, the DWM here average 30bp in length (the ideal length of the flank is probably factor-specific, and has not been investigated in detail here). A DWM is also harder to visualise: a “sequence logo” cannot capture correlations. While one can consider a representation of “conditional” sequence logos resulting from fixing particular nucleotides, the result would be unwieldy and not very informative. I argue that PWMs and DWMs can live together (just as “consensus” sequence strings continue to be widely used despite the invention of sequence logos). PWMs have their utility as a concise and easy representation of binding motifs, while DWMs offer much better precision in prediction.

## Results

### Correlations of gapped dinucleotides, and gap distribution

The first question to be answered is whether going beyond PWMs is important enough to justify the additional complexity of DWMs. We examine 40 transcription factors in yeast (that are further studied in the benchmarks below), each of which has at least 32 predicted targets in MacIsaac *et al.* [20]. For each of the predicted target sequences, the PWM supplied by MacIsaac *et al.* was used to predict the best binding sites, plus any additional binding sites with a log-odds of greater than 3.0. For each factor, all pairs of positions within the binding sites were examined for dinucleotide correlations.

Let 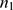, 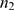 be two positions within the binding motif, with 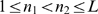, where 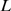 is the length of the motif. Let there also be 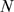 binding sequences in total. We also construct a position weight matrix 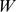 using these 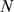 sequences. For each pair of positions, there are 16 possible dinucleotides 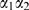, each of which is examined. If the PWM hypothesis of position-independence holds, the expected number of sequences containing the nucleotides 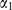 at 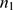 and 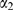 at 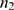 will be 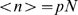, where 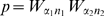 is the probability of that dinucleotide. The standard deviation will be 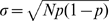. Let 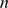 be the number of sequences that actually contain this dinucleotide. In the following, we consider 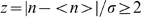 to be evidence of significant dinucleotide correlations. For normally distributed data, fewer than 0.05% of the data points should differ by more than two standard deviations from 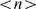.

It turns out that out of 1,734 dinucleotide-pair positions studied, 322 deviate from the independent-nucleotide assumption by 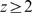. However, a large number of these cases involve extremely small PWM probabilities, and the number of sequences containing these dinucleotides is rather low (but the expected number, and the expected variance, are both close to zero). Therefore we additionally require that 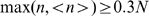; this yields 87 dinucleotides, still greater than the unrestricted number of correlated sequences expected by chance.

**Figure 1:**
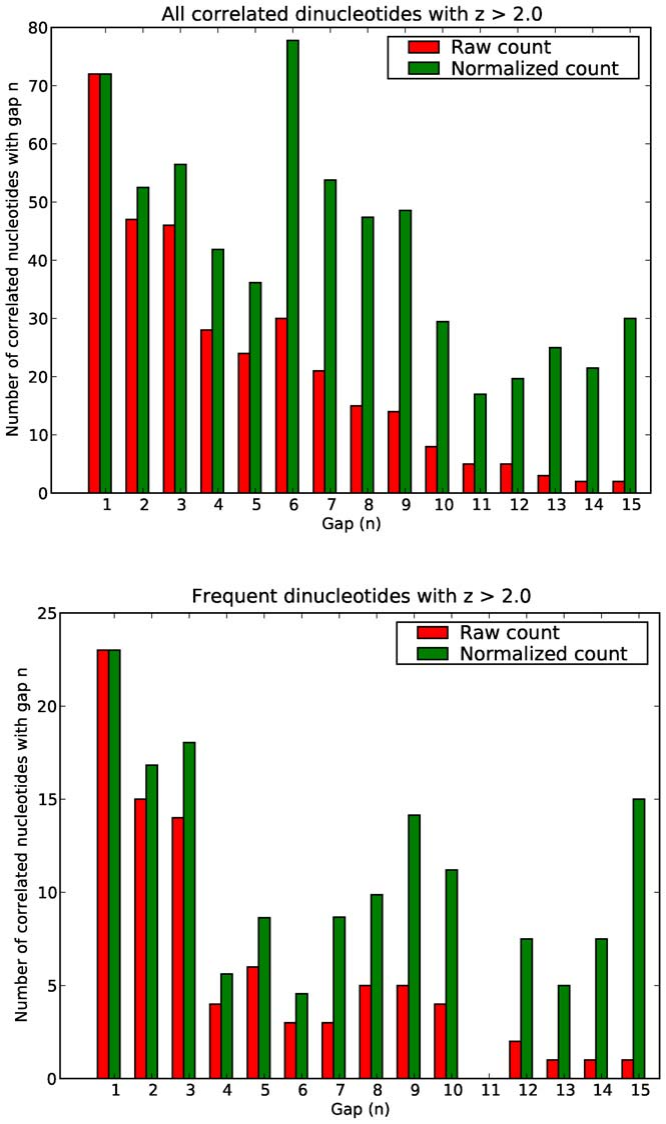
The distribution of gaps in correlated dinucleotide pairs (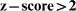) in yeast TFs, as described in the text. The graph on top shows the full distribution, and the graph below shows only those pairs that are sufficiently abundant (either the predicted or actual number being at least 30% of the total). The green “normalised” bars include a correction for there being fewer possible pairs with larger “gaps”. With this correction, the graphs are more uniform.

The next question is how the gaps in these dinucleotides are distributed. Figure 1 shows the answer: while nearest-neighbour dinucleotide correlations are the most common, dinucleotide correlations are found at all spacings. Moreover, the dominance of short-ranged correlations is partly explained by the fact tha there are more short-range pair positions (for a motif of length 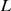, there are 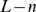 dinucleotides separated by a “gap” 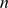 (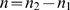, above). Correcting for this produces a somewhat more uniform distribution of gaps, up until roughly 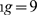, after which occurs a fall-off. This, fall-off, too, is perhaps explained by the fact that there are fewer factors with long binding motifs.

Detailed examination reveals a few other points: in most cases, 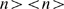, that is, there are more dinucleotides seen than would be expected from the PWM values at those positions. Some factors deviate more from PWM values than others, and in many cases, these are the same factors that perform well in the yeast benchmark below, as described there. For details of all factors and deviating column pairs, see Text S1.

### Benchmarks for the DWM method

Two sets of benchmarks are described below: a large benchmark on yeast data, using 40 transcription factors, and a smaller one on fruitfly data, using the *hunchback* transcription factor. In both cases, predictions from the position or dinucleotide weight matrices that we construct are compared, and compared with previously available (“prior”) position weight matrices.

The prior PWMs were used “as is”, but in constructing our PWMs and DWMs, sites from the target sequence being benchmarked were excluded. This is important since, when the number of sequences is relatively small, such “self-prediction” can significantly affect the results, especially in the dinucleotide case.

### Binding site predictions in yeast

These benchmarks use the genome-wide binding data from ChIP-chip experiments reported by Harbison *et al.* [19] and the revised predicted targets reported by MacIsaac *et al.* [20]. For 40 transcription factors that had at least 32 predicted high-confidence targets, we constructed new PWMs and DWMs, with and without 10bp flanks, as described in Materials and Methods. The matrices were constructed using predicted sites in the targets, but as observed above, “self-prediction” was avoided. Therefore, if there were 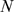 targets, 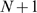 matrices were constructed: one that used all targets as data, and one omitting sites from each target by turn, to be used in predicting sites for that target. The prior PWMs, constructed PWMs, and constructed DWMs were used to predict binding sites on all sequences from the original ChIP experiments. The results were compared with the raw binding “p-values” for the same sequences reported by Harbison *et al.*, as well as with the predicted targets from MacIsaac *et al.*

**Figure 2:**
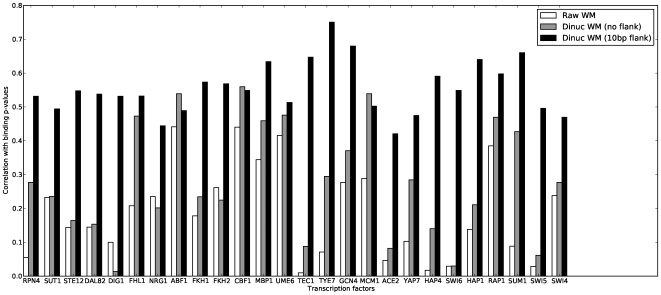
The relative performance of PWMs and DWMs in predicting binding targets in yeast. The figure shows Pearson correlation coefficients of binding site predictions with ChIP binding 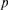-values reported by Harbison *et al.* [19], using the “raw” position weight matrices from MacIsaac *et al.* [20], dinucleotide weight matrices with the same “width” as the “raw” matrices, and dinucleotide weight matrices with a 10bp “flanking sequence” on either side of the input matrices. Details are in Materials and Methods.

Figure 2 shows the Pearson coefficient of correlation with binding data in Harbison *et al.* [19]. The calculation is described in Materials and Methods. This figure only shows those 26 factors for which predictions correlate with a coefficient of at least 0.3 for at least one of the three methods shown (the original PWM, our DWM without flank, or our DWM with flank). In nearly all of these cases, the dinucleotide matrix, and in particular the DWM that includes flanking sequence, greatly outperforms the PWM. Data for all the factors, and also for our “posterior” PWMs, are portrayed in Figure S1.

One may ask whether the improved coefficient of correlation is merely a consequence of the fewer predictions made by the DWMs. To answer this, Supporting Figure S2 shows (for all 40 factors) the coefficient of correlation for the top 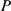 predictions from the prior PWM, where 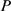 is the number of predictions made by the DWM with 10bp flank, plus any further predictions with the same logodds as the lowest in this set. In many (but not all) cases, the correlation coefficient is improved; however, in most cases, it remains well below what is achieved by the DWM.

**Figure 3:**
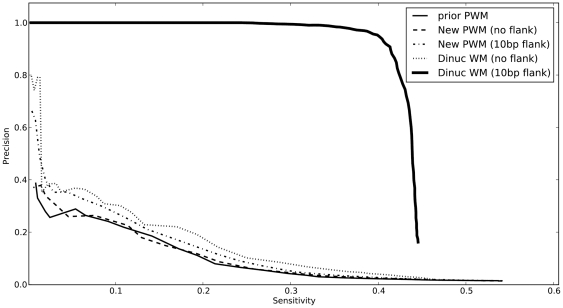
The precision, as a function of sensitivity, of PWMs and DWMs in predicting targets from MacIsaac *et al.* [20]. The precision is the fraction of predictions above a certain logodds cutoff 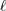 that correspond to documented target genes. The sensitivity is fraction of known targets that are predicted above that cutoff. These are for the same benchmark data as in Figure 2.

Figure 3 shows the “precision” of predictions for the annotated target genes [20], that is, the fraction of predictions at or above a given logodds cutoff 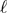 that are listed as a target, as a function of the sensitivity to known targets, that is, the fraction of listed targets that are found at or above the logodds cutoff 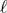. The prior and new position weight matrices, without flanking sequence, perform very similarly. While either adding flanking sequence alone, or using a dinucleotide matrix alone, cause notable improvements (the dinucleotide WMs without flank are about 50% to 100% more specific than the prior PWMs), DWMs with flank achieve nearly perfect precision over most of the range of sensitivity. Note that the precision here refers to gene target, not to individual binding sites.

**Figure 4:**
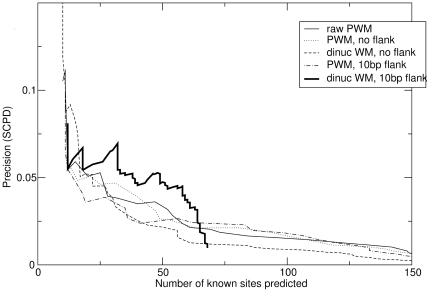
The performance of different methods on individual site predictions in yeast. For the same benchmark as in Figure 2, these are the fraction of site predictions that agree with annotated sites in SCPD, as a function of the total number of SCPD sites predicted.

To measure sensitivity to individual binding sites, we combined these data with the *Saccharomyces cerevisiae* Promoter Database (SCPD) [21]. 19 of the 40 factors that we consider contain annotated sites in SCPD. Figure 4 plots the fraction of site predictions for these 19 factors that are annotated in SCPD (“precision” to SCPD), as a function of the total number of SCPD sites predicted. Since SCPD is far from an exhaustive database, false positives cannot be counted, but these “precision” curves are hopefully reflective of the true precision if all true binding sites were known.

Finally, we observe some interesting points about specific factors. For each factor, if we look at the number of column pairs that are more than 2 standard deviations away from the PWM expectation (Results, first subsection), and also ask that either the expected or the observed number of dinucleotides is 30% of the number of sequences, we find (as noted earlier) that, across all factors, there are 87 correlated column pairs. Looking at individual factors, we find that there are 10 factors that have 3 or more correlated column pairs, namely RPN4, FKH2, CBF1, ABF1, DIG1, HAP4, TEC1, SUM1, STE12 and MCM1 (which has a remarkable 19 column pairs showing significant correlation). Comparing with Figure 2, we find that for eight of these factors the DWM method greatly outperforms the PWM method: the exceptions are ABF1 and CBF1.

Maerkl and Quake [7] studied the basic helix-loop-helix factors PHO4 and CBF1, together with two human factors, and argued that PWMs are insufficiently able to discriminate while providing many false positives. While PHO4 is not in our list (having only 23 predicted high-confidence targets) and DWMs do not perform notably better than PWMs for CBF1, it is notable that in the case of another HTH factor with a similar binding motif (TCACGTG), TYE7, PWM predictions correlate very poorly with binding data while DWM predictions correlate nearly perfectly. Similarly, ACE2 and SWI5, homologous factors [22] which share similar binding motifs (GCTGGT), are much better discriminated by DWMs than by PWMs; as, to a lesser extent, are MBP1 and SWI4, which are homologous cell-cycle-related proteins [23]. In many of these cases, including flanking sequence improves the results, suggesting that flanking nucleotides show significant correlations with nucleotides within the core motif, or with one another.

### Binding site predictions in fruitfly

The REDfly (formerly FlyReg) database [24], [25] contains curated DNAse I footprints of binding sites for several transcription factors in *Drosophila melanogaster*. These form a useful resource for benchmarking, but since a rather small fraction of functional sites are likely to be annotated in this database, the benchmark here uses synthetic sequence that contains embedded REDfly footprints as well as synthetic samples from PWMs, as described in Materials and Methods. The goal was to predict the functional sites, and also to discriminatively predict the functional sites rather than the synthetic samples, using PWMs and DWMs.

Several ChIP-on-chip experiments for transcription factors in *Drosophila melanogaster* have been reported in the literature. Here, data from Li *et al.* [26], who studied six factors, are used. For reasons explained in Materials and Methods, I focussed on the factors *bicoid* (*bcd*), *hunchback* (*hb*), and *kruppel* (*Kr*). Using prior PWMs from B1H data in [27], binding sites were identified in the ChIP peaks and used to construct PWMs and DWMs, with and without a 10bp flank. Peaks that overlap with REDfly footprints were carefully excluded, for the reasons noted earlier.

The results for the *hunchback* factor were impressive. The binding motif for this factor is a weak poly-A pattern that is abundant in the genome; it appears that the dinucleotide method in this case significantly improves the precision of predictions, and, as in the case of the yeast factors, flanking sequence plays a role.

With the other factors (*bicoid* and *kruppel*), the dinucleotide method did not show improvement over the PWM method (data not shown), and in fact, in the case of *bicoid*, the input (B1H-derived) PWM showed significantly better precision in predicting REDfly footprints than even the ChIP-derived PWMs. The reasons are unclear, but a more thorough study of *Drosophila* factors is in progress. Meanwhile, *kruppel* binds to a relatively sharp and well-defined motif, so it is possible that there is no important additional information in dinucleotide correlations.

**Figure 5:**
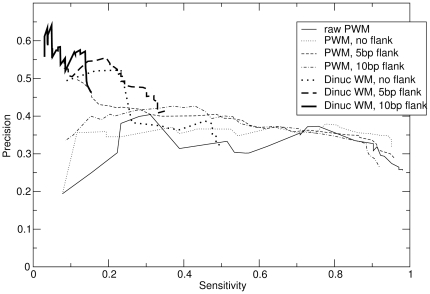
The precision of site predictions in fruitfly. For predictions in synthetic sequence embedding binding site footprints from the REDfly database as well as “fake” sites that are samples of PWMs corresponding to the same factors, this plot shows the precision in predicting REDfly sites, that is, the fraction of predictions that overlap with REDfly footprints, as a function of sensitivity, that is, the fraction of real (REDfly) sites that are predicted. Details of the construction of the synthetic sequence are in Materials and Methods.

**Figure 6:**
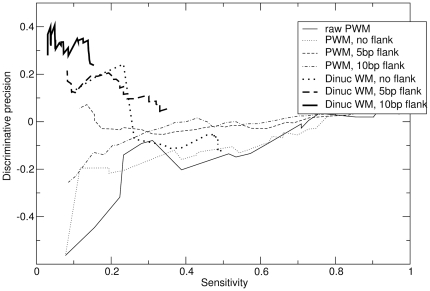
The *discriminative* precision of predictions in fruitfly. For the same predictions as in Figure 5, this plot shows the “discriminative precision” for REDfly sites, that is, difference in the fraction of predictions that overlap with REDfly footprints and the fraction of predictions that overlap with “fake” sites, as a function of sensitivity.

Figure 5 plots the precision of *hunchback* predictions for real (REDfly) footprints. Figure 6 plots the “discriminative precision”. Here the precision is defined as 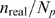 and the discriminative precision is 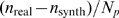, where 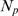 is the total number of predictions above a particular logodds cutoff, 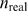 is the number of predictions that overlap real (REDfly) footprints, and 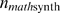 is the number of predictions that overlap sites that were sampled from the PWM, as a function of the “sensitivity”, the fraction of real REDfly footprints that are overlapped by predictions above the same cutoff. Unlike in the yeast SCPD benchmark, these sites are embedded in synthetic sequence; therefore any prediction that is not a REDfly footprint can safely be termed a “false positive”. Given the variability of TF binding widths as well as REDfly footprints, and also given the large size of many of the REDfly footprints, predictions whose midpoint lay within 10bp of the REDfly footprint were considered “hits”.

The results suggest that the precision of dinucleotide-model predictions is substantially better than PWMs, for a given sensitivity, and for high-confidence predictions DWM predictions are nearly twice as specific to REDfly sites as PWM predictions. But the sensitivity of dinuc WMs is substantially less than PWMs, especially when flanking sequence is included. With the “discriminative precision” the difference is even sharper: PWM predictions mostly have negative discriminative precision, that is, they resemble synthetic samples from themselves more strongly than they resemble actual binding sites; and while the discriminative precision of DWMs gets better for higher-confidence predictions, PWMs actually perform worse in this regard. For *hunchback*, then, DWMs with flanking sequence are clearly better able to distinguish genuine binding sites from similar sequences generated as samples from the respective PWMs.

## Discussion

The benchmarks on ChIP-characterised factors in yeast and fruitfly suggest strongly that the DWM provides a much-improved representation of binding sites for many transcription factors. In a way, this is unsurprising: DWMs contain, in the worst case (the case of completely uncorrelated positions in a motif), the same information as PWMs, and in other cases much more information; and this must be reflected in their predictive power. The DWMs here were constructed excluding sites from target sequence: therefore, it is reasonable to assume that “complete” DWMs will perform even better. The important points are that, first, the approximation to the likelihood in equation (10) is useful and works well here (its usefulness outside this narrow context remains to be explored, but one can be optimistic); second, a few dozen known binding sites are sufficient to arrive at a reasonably high-quality DWM; third, flanking sequence appears to play a significant role, that is not so strongly apparent when using PWMs.

This approach is thus very promising for the future. While the starting point of an investigation may be a PWM based on a few binding sequences, such a PWM combined with possibly noisy genome-wide binding data may perhaps be used to “bootstrap” a DWM representation. That DWM may in turn be used to predict more binding sites, with much greater confidence than a PWM can ever do.

However, for some factors in yeast, and for *bicoid* and *kruppel* in fruitfly, DWMs seemed to not perform better than PWMs, or even performed worse. The reasons need to be understood, but it may simply be inadequate prior binding data in some cases. Further work is in progress on *Drosophila* factors.

This paper uses a naive method for predicting sites: the log-odds for the binding sequence being explained by a PWM or DWM over a background model. Better methods are commonly implemented with PWMs, for example, using biologically-motivated prior binding probabilities; and taking account of competition between different factors (for example, Stubb [28], a *cis*-regulatory module prediction program). In principle, all the same improvements can be applied to DWM predictions.

It would be of great interest to relate DWMs with a more biophysical binding-energy model of protein-DNA interactions. Just as PWMs can be derived from a simple binding-energy model with some additional assumptions ([10], supporting text), DWMs should be justifiable in terms of protein-DNA binding energetics. As noted earlier, non-independence of nucleotide distributions at different positions probably implies non-addititivity of the binding energy, and this should be taken into account in building improved models.

*Ab initio* motif-finding and prediction of binding sites using DWMs, and the usage of homologous sequence from related species to improve predictions, are interesting topics that deserves to be addressed in the near future, perhaps as extensions of the PhyloGibbs program [29], [30]. Predicting *cis*-regulatory modules using this formalism would also be a useful and interesting exercise.

In summary: The dinucleotide weight matrix described here is easy to calculate, though cumbersome. The method described here of calculating posterior probabilities of binding sites is straightforward, though approximate. When large numbers of binding sites are already known, this formalism should be preferred to PWMs in predicting new sites.

However, it should be emphasised that the DWM formalism presented here is subject to further modification and refinement. In particular, the question of the appropriate “pseudocount” to apply to DWMs is not easy and the answer here is by no means definitive. The appropriate length of flanking sequence is probably highly factor-specific. Lusk and Eisen [31] recently observed that the “cutoff score” used to imply significance for PWM-based binding site predictions is probably variable across factors, and the same will certainly be true for DWM-based predictions. Therefore, while DWMs represent a significant advance over PWMs in predictive power, a “one-size-fit-all” solution to the problem of binding-site prediction is unlikely to exist.

### Implementation and availability

All benchmarks listed here were performed using scripts written in Python by the author. These are not user-friendly but are available, with some basic documentation, from the author for interested users. The DWMs generated for the factors discussed in this paper are available as Python “pickle” dumps (which can be loaded and used by other Python programs). A user-friendly, fast implementation of these methods in a compiled language is planned in the future.

## Materials and Methods

### Constructing PWMs and DWMs from binding-site data

Given 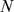 known aligned binding sequences, a PWM can be constructed with normalised base counts in these sequences: in column 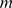, let there be 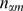 instances of the nucleotide 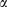, with 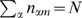. Then, for 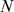 large, 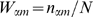. Usually 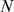 is not terribly large, so one instead uses
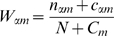
(2)where 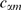 is a “pseudocount” and 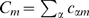. Formally, this is the same as assuming a Dirichlet prior on 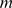
′th column of the weight matrix, 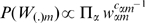. (See the book by Durbin *et al.* [32] for a discussion.) The special choice 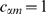 expresses complete prior ignorance of 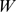, and is generally appropriate for estimating weight matrices.
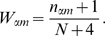
(3)We use this choice to construct our PWMs, both for direct benchmarking and for use in the DWM formulas derived below.

If we were completely ignorant of dinucleotide probabilities, we should use the analogous expression to construct DWMs:
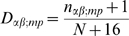
(4)where 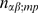 is the number of sequences where nucleotide 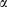 is found at column 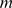 and 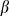 at column 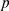. But we know that, in practice, different columns tend to be roughly independent (that is, PWMs generally work well); and for a given 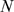 we have a much better estimate of the PWM 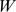 than of the DWM 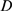. Therefore, instead of the pseudocount 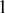 that implies complete ignorance, we use as our prior the product of the corresponding PWM columns, normalised to sum to 16:
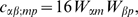
(5)

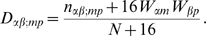
(6)Other choices of priors and pseudocounts are, of course, possible, but the choices above are straightforward and work well.

### Using the DWM to calculate posterior probabilities

We would like to calculate 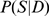, that is, the probability that a putative binding sequence 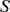 is “explained” by a dinucleotide model 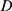. (We can compare it to 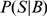, the probability of it arising from a “background model” 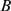; the ratio of these is the “odds”, and the logarithm of this ratio is the “log-odds”.)

First we write
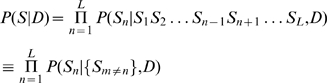
(7)that is, the probability of observing the sequence is the product of the probabilities of each nucleotide 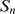 given all the other nucleotides 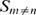 in the sequence, and given the dinucleotide model 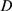. (This is an approximation: the sum of this over all sequences will not be exactly 1, though the sum of each factor over 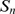 is 1. However, since we use it essentially as a discrimination score, we ignore this matter). We estimate these individual nucleotide probabilities using the Bayesian expression
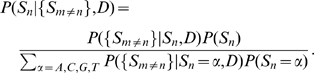
(8)Here, for the prior probabilities 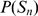 and 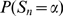 we use the single-nucleotide weight matrix values 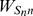 and 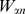. Finally, we approximate the likelihood of neighbouring sequence given the nucleotide 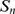 as the product of individual conditional probabilities:
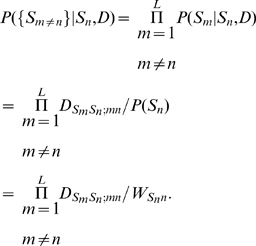
(9)That is, we write this likelihood as a product of conditional probabilities of the individual nucleotides 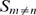 given 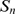; these conditional probabilities are evaluated in the usual way, 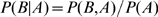. Putting all of this together, the final expression for 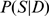 is
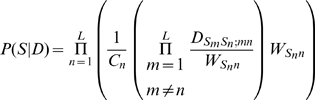
(10)where 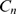 is a normalisation constant for the 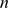
th factor in the product (equal to the denominator in equation 8). In the case that there are no dinucleotide correlations, we have 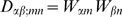 for all 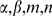, and the expression reduces to the PWM-based answer, 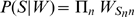.

### Yeast binding site prediction benchmarks

Of the factors studied in the ChIP-on-chip benchmarks reported in Harbison *et al.* [19], 40 factors were selected that had at least 32 targets annotated in MacIsaac *et al.* [20], with a 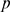-value of 0.001 or better and conservation in 2 species (filename orfs_by_factor_p0.001_cons2.txt), and with a corresponding sequence (or sequences) in the microarray probe file (filename yeast_Young_6k.fsa). Prior matrices were taken from supporting data of MacIsaac *et al.* (filename v1.tamo). Raw 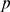-values for binding were taken from supporting data of Harbison *et al.* (filename Harbison_Gordon_yeast_v9.11.csv). All these files were downloaded from the supporting data pages hosted by the authors of those papers. The prior matrices are in logodds format, in most cases using genomic single-nucleotide frequencies for the background model; they were converted to position weight matrices that give the probabilities of individual nucleotides.

The posterior PWMs and DWMs were constructed by the following two-step process: first, for each predicted target, the highest-scoring sites were selected using the prior PWM, with the following criterion: all sites with a logodds of above 3.0 (natural logarithm) were selected. If there were none, but the best site had a logodds of at least 1.5, that site alone was selected. If there were no good matches, the sequence was rejected. These putative binding sequences were aligned (with or without a flanking sequence of 10bp) and used to construct new, interm PWMs and DWMs. These DWMs and PWMs, were in turn used to predict sites in all targets, using the same logodds criteria as earlier (with no additional flanking sequence). The resulting predictions were aligned to construct the final “posterior” PWMs and DWMs, with one difference: in addition to “full” PWMs and DWMs, “partial” PWMs and DWMs were also constructed for each contributing probe by omitting all binding sequences from that probe, and these partial matrices were used for the predictions in that probe described below, in order to ensure that all predictions were based on matrices of completely independent origin.

For constructing the PWMs and DWMs, I chose to use predicted sites, rather than experimentally validated sites, because there are not sufficient numbers of the latter available for most factors. While a genome-wide PWM-based or DWM-based bioinformatic search for binding sites is likely to pick up many false positives, we argue that if we confine the search to regions that are predicted by ChIP experiments to be bound, with high confidence, to the TF in question, and only select the most likely predictions in these regions, these are much less likely to be false positives, while also being much more numerous than experimentally validated sites in databases such as SCPD. Also, while a genome-wide PWM-based search is unlikely to result in positional correlations within predicted sites, such correlations are arguably more likely when only predictions in ChIP-validated regions are considered; and the “bootstrapping” procedure of using the initial DWMs to predict a new set of sites should result in further refinement. These remarks also apply to the methods used for *Drosophila* factors described below.

These PWMs and DWMs, as well as the prior PWMs, were then used to predict sites in every probe sequence in the microarray probe file. To construct Figure 2 and Figure S1, for each method, the total logodds prediction for each probe sequence was calculated (that is, the logodds at each site was summed over all possible sites, with the better of two “orientations” chosen at each site): this was done in order to treat equally the cases of a factor having a few highly specific sites, or several weaker sites. This was cross-correlated with the geometric mean of the “rich medium” 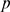-value and the (up to) two best other 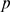-values in the file cited above. Up to two other values were averaged because there could be cases where a TF does not bind strongly in the default “rich medium” condition but does bind more strongly under certain other biological conditions, for reasons that cannot be predicted in this sort of bioinformatic analysis. However, if binding was not reported in at least two other conditions, fewer than two other 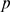-values were averaged. The Pearson correlation coefficients (with the probe as independent data, and the “total logodds” and “mean 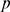-value” as dependent data) are plotted. The calculation is over all probes.

Figure 3 was plotted using the same data, as described in the main text.

The SCPD database includes binding data for 234 factors/complexes in yeast, of which 19 were common to the list of 40 factors studied above. 208 binding regions were annotated for these 19 factors. I extracted these regions, converted them to genomic coordinates, and analysed the precision of the previous site predictions for these factors of the PWM and DWM methods to these sites, as a function of the number of known sites recovered. For this purpose, since the SCPD coordinates are widely variable in size (some “sites” are only one nucleotide long), and the PWMs and dinucleotide WMs are also of different sizes, the following criterion was used: if the midpoint of the annotated SCPD region was within 10bp of the midpoint of the predicted binding site, the region was considered successfully predicted.

### Fly binding site prediction benchmarks

The ChIP data used here were taken from Li *et al.* [26]. In this preliminary investigation, to ensure high confidence in predictions, those factors were taken that were bound by 2 antibodies: namely, *hb*, *bcd*, *kni* and *Kr*; and only peak positions overlapped by both sets of antibodies were considered. This yielded 83 peaks for *bcd*, 230 for *hb*, and 818 for *kr*, but only 12 for *kni*. The latter was accordingly dropped and the former three used. Footprints for all of these were obtained from the REDfly database [25], and any footprints that overlapped with the peak list were removed from the peak list. Prior PWMs were obtained from the B1H study of Noyes *et al.* [27], and used to construct posterior PWMs and DWMs in the same two-step manner as described in the yeast benchmark.

The benchmark was on synthetic sequence in which the actual REDfly footprints for each factor, plus 10bp flanking sequence, were embedded. These footprints, with flanking sequences, were separated by 100bp random spacer sequences. In addition, synthetic sequence of the same length, but containing embedded samples of PWMs rather than actual REDfly footprints, was included. The number of copies of synthetic sites was the same as the number of copies of real sites, for each factor. Predictions where the centre of the prediction lay within 10bp of the footprint region were considered “hits”.

## Supporting Information

Text S1 Details of significant dinucleotides in yeast TFs.
(0.11 MB TXT)

Figure S1 Details of performance of PWMs and DWMs on yeast TFs. Pearson coefficients of correlation for logodds predictions with published binding p-values for all 40 factors studied, for all matrices used (prior PWM, posterior PWMs and DWMs with and without flanking sequence). Also shown is the correlation for prior PWMs when only the top N are considered, where N is the number of predictions from the DWM with flanking sequence, plus any additional predictions with an equal log-odds score. In addition, sequence logos are shown for the prior PWMs and the posterior PWMs with flanking sequence, in both orientations. In most cases, the logos are extremely similar and there is little sequence signature in the flanking sequence at the PWM level.
(1.70 MB PDF)
