# Supplementary figures and images for: Dinucleotide Weight Matrices for Predicting Transcription Factor Binding Sites: Generalizing the Position Weight Matrix
Source: PLoS One. 2010 Mar 22;5(3):e9722. doi: 10.1371/journal.pone.0009722 (PMC2842295; doi:10.1371/journal.pone.0009722)

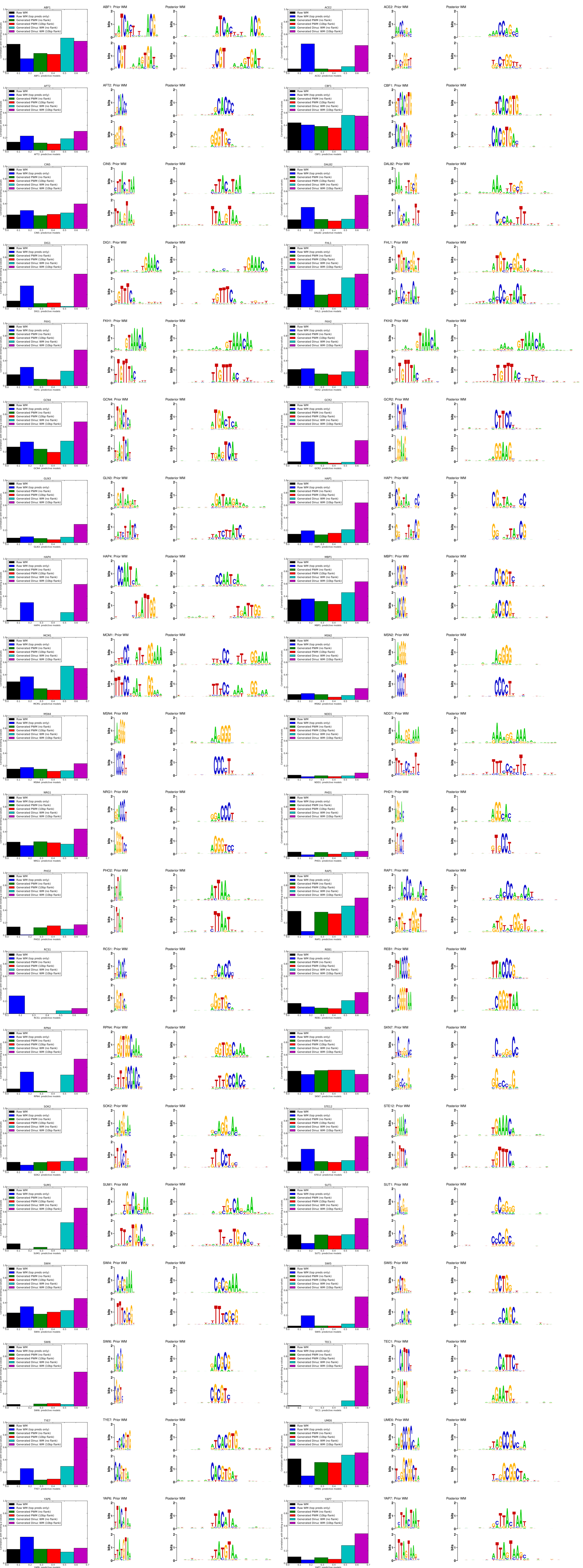

Supplement: Figure S1 — Details of performance of PWMs and DWMs on yeast TFs. Pearson coefficients of correlation for logodds predictions with published binding p-values for all 40 factors studied, for all matrices used (prior PWM, posterior PWMs and DWMs with and without flanking sequence). Also shown is the correlation for prior PWMs when only the top N are considered, where N is the number of predictions from the DWM with flanking sequence, plus any additional predictions with an equal log-odds score. In addition, sequence logos are shown for the prior PWMs and the posterior PWMs with flanking sequence, in both orientations. In most cases, the logos are extremely similar and there is little sequence signature in the flanking sequence at the PWM level. (1.70 MB PDF) [file pone.0009722.s002.pdf]
